# Phylogeny and genetic structure in the genus *Secale*

**DOI:** 10.1371/journal.pone.0200825

**Published:** 2018-07-19

**Authors:** Öncü Maraci, Hakan Özkan, Raşit Bilgin

**Affiliations:** 1 Department of Animal Behaviour, Bielefeld University, Bielefeld, Germany; 2 Evolutionary Biology, Bielefeld University, Bielefeld, Germany; 3 Institute of Environmental Sciences, Boğaziçi University, Istanbul, Turkey; 4 Department of Field Crops, Faculty of Agriculture, University of Çukurova, Adana,Turkey; National Cheng Kung University, TAIWAN

## Abstract

*Secale* L. is a small but important genus that includes cultivated rye. Although genetic diversity of cultivated rye is high, patterns of genetic diversity in the whole genus, and potential factors affecting the distribution of genetic diversity remain elusive. The population structure and distribution of genetic variation within *Secale*, and its correlation with taxonomic delimitation, cultivation status or spatial distribution in relation to geography and climate zones were analyzed in this study. A collection of 726 individual plants derived from 139 different accessions representing *Secale cereale*, *S*. *vavilovii*, *S*. *strictum*, and *S*. *sylvestre* were investigated using SSR analysis and sequence diversity analysis of a nuclear EST region. Our results indicated that perennial *S*. *strictum* subspecies are genetically divergent from annual forms of the genus. Existence of two distinct clusters within the annual taxa was observed, one corresponding to samples from Asia, and a second to those outside of Asia. No clear genetic structure was observed between different annual species/subspecies, indicating introgression between these taxa. The analysis of cultivated rye revealed that landrace populations from the Middle East have the highest genetic diversity, supporting the idea of the area being the center of origin for cultivated rye. Considering high adaptive potential of those populations, Middle Eastern landraces should be regarded as genetic resources reservoirs for new niches and future breeding programs.

## Introduction

Sustainable food production is a vital environmental issue, in the context of global climate change. Elevated temperatures and accompanying alterations in precipitation regimes are expected to decrease yields significantly. At the same time, global requirement for food is expected to increase by 60% by 2050. The adaptive capacity of plant populations under stress conditions are positively related to the degree of genetic diversity maintained in those populations [1]. Genetic diversity of modern varieties (cultivars) of crop plants is quite low due to genetic erosion stemming from domestication syndrome and modernization bottlenecks. On the other hand, wild relatives of crop plants and unimproved varieties known as ‘landraces’ are genetically diverse [2,3] and contain many adaptive alleles in their gene pools.

*Secale* L. is a small but economically important taxon, which includes such wild relatives and landraces, comprising cultivated rye, containing annual, perennial, self-incompatible or self-compatible, cultivated, weedy and wild taxa [4]. Although the genus is regarded as the typical representative of Mediterranean flora and Southwest Asia, specifically Turkey, Lebanon, Syria, Iran, Iraq, and Afghanistan are the main centers of its distribution [5]. Taxonomy of the genus *Secale* is still not without contention, due to disagreements on the delimitation of species and intraspecific taxa, the out-crossing nature of many species, and lack of hybridization barriers between species and subspecies. Hence, regarding the taxonomical classification and phylogeny of *Secale* species, several hypotheses were proposed [5–8]. Recent molecular findings [9–11] are consistent with Frederiksen and Petersen`s [7] hypothesis that the genus is represented by three species: *S*. *sylvestre*, *S*. *strictum* and *S*. *vavilovii*. There is a general agreement on *S*. *strictum*, the perrenial species being the ancestral form [10,11]. The first species that diverged from *S*. *strictum* was proposed to be *S*. *sylvestre* [9,12,13]. *S*. *strictum* subsp. *africanum* is considered to have diverged from *S*. *strictum* during the early Pleistocene and evolved independently [13]. *S*. *cereale* and *S*. *vavilovi*, on the other hand, are considered to be evolutionarily the youngest species [12,14]. Although *S*. *vavilovi* was classified as a distinct species based on morphological differences [6,8,15], recent molecular studies revealed no clear difference between *S*. *vavilovii* and *S*. *cereale* [10,11,13,16], and suggested that *S*. *vavilovii* should be ranked as a subspecies within the *cereale* group.

For the ancestry of cultivated rye, *S*. *cereale* subsp. *cereale*, different researchers have different opinions as well: *S*. *vavilovii* [17,18], *S*. *ancestrale* [19], and *S*. *strictum* [20] were suggested to be the progenitor of cultivated rye. Similarly, the exact geographical center of origin for cultivated rye is not known, but south-western Asia was proposed to be most probably the center of origin [5,18]. Although production of cultivated rye declined worldwide during the 20^th^ century, it has long been an important crop in Northern and Central Europe, especially in the cooler parts that is not suitable for cultivation of other cereals [21]. Rye has a cross-pollinating reproductive system and thus its levels of intraspecific diversity are high compared to self-pollinating grains [22]. Although, cultivated varieties of cereal rye has been experiencing extensive genetic bottleneck [23] due to strong selection pressure, like many other cereal crops, significant proportion of genetic diversity is maintained in landraces [24,25] and wild and weedy forms [26,27]. Furthermore, these populations contain potentially useful traits such as resistance to diseases, adaptability to biotic and abiotic stress [28,29]. Therefore, considering that the wild and weedy forms may crossbreed with cultivated rye [30], these taxa, along with the landraces, constitute gene pools for desirable genes, and can be regarded as genetic resource reservoirs for new niches and future breeding programs of wheat, triticale and other crops [22]. Hence understanding genetic structuring of the genus *Secale* and distribution of genetic diversity within the genus is extremely important. In this vein, with this study, we investigated wild, weedy, landrace and cultivated varieties of rye using Simple Sequence Repeats (SSRs) and sequence diversity analysis of a nuclear Expressed Sequence Tag (EST) region in order to obtain further insights about taxonomy, phylogeny, and genetic structure of *Secale* species. Specifically we evaluated the correlation of genetic structure with taxonomic delimitation, cultivation status or spatial distribution in relation to geography and climate zones [31] at a global scale.

## Materials and methods

### Plant material and DNA extraction

In this study, a total of 726 samples belonging to 139 different accessions of cultivated varieties, landraces, weedy and wild populations of *Secale* were investigated from 45 different countries (S1 Table). Among these, 584 samples from 100 accessions of *S*. *cereale*, 46 samples from nine accessions of *S*. *vavilovii*, 89 samples from 23 accessions of *S*. *sitrictum*, two samples from two accessions of *S*. *sylvestre*, and five hybrid samples were used (S1 Table). In terms of cultivation status, 137 genotypes belonged to wild accessions, 51 genotypes to weedy accession, 343 genotypes to landrace accessions and 195 genotypes to cultivated accessions.

The accessions were provided by United States Department of Agriculture Germplasm Resources Information Network (USA), and Leibniz Institut für Pflanzengenetik und Kulturpflanzenforschung (Germany). Two accessions were collected in farms from Turkey, in 2010. In order to confirm taxonomic delimitations of accessions, seeds were planted in trial fields from December 2010 to June 2011, and the samples were regularly evaluated for certain phenotypic characters during all developmental stages following [7]. Total DNA was extracted according to the method described by Doyle and Doyle [32].

#### SSR analysis

Initially, 20 nuclear SSR primers previously used in the genus *Secale* [33,34] were screened in eight individual plants, representing four *Secale* species in terms of PCR amplification success and peak profiles. Among these, a set of ten microsatellite primers yielding good PCR products and scorable peaks were selected (REMS1187, REMS1254, REMS1323, REMS1264, REMS1205, REMS1238, REMS1160, REMS1303, REMS1259 and SCM 180) and used for the analysis of 721 samples (S2 Table). All PCR reactions were performed as described by Khlestkina et al. [33] and Saal and Wricke [34]. Amplification success was checked and successful PCR products were read on an ABI 3100 capillary sequencer with GS400HD size standard (Applied Biosystems).

The alleles were automatically binned using FlexiBin [35] and checked manually. The genotyping errors stemming from null alleles, large allele dropout or the scoring of stutter peaks that can potentially lead to deviations from Hardy–Weinberg proportions were detected using Micro-checker version 2.2 [36]. Based on the results, three markers (REMS1303, REMS1259 and SCM 180) were and seven SSR markers (REMS1187, REMS1254, REMS1323, REMS1264, REMS1205, REMS1238, REMS1160) were used for the subsequent analyses. The mean polymorphism information content (PIC) was calculated for each marker using MolKin v.3.0 software [37].

We analyzed the whole data set, consisting of 721 samples, excluding the hybrids, in three different categories on the basis of (1) taxonomic identity, (2) cultivation status and (3) climatic conditions of geographical origin. In the first category, all of the genotypes were pooled into 11 groups based on their taxonomic identity, at the species and subspecies level, in order to understand the distribution of genetic diversity in different taxonomic groups. In the second category, all genotypes were grouped as wild, weedy, landrace and cultivated varieties, to evaluate the effect of cultivation status on the distribution of genetic diversity. In the third category, all genotypes excluding two samples of unknown geographical origin were assigned to 18 climate subgroups belonging to five main climate groups, as determined by Köppen-Geiger classification system, which is based on classifying the mean climate conditions on geographic areas around the globe using different climatic variables [31], in order to understand whether climatic conditions of geographic origins affect distribution of genetic diversity.

In addition to these three categories, patterns of genetic diversity in cultivated rye *i*.*e*. *S*. *cereale* subsp. *cereale* genotypes (both landrace and cultivated varieties were analyzed separately. In this analysis, a total of 533 genotypes from 83 different accessions originating from various geographical regions, representing 10 main gene pools (Africa, Australia, Europe, Balkans, Caucasus, East Asia, South and Central Asia, Middle East, North America and South America) were used. These samples were analyzed using the same seven microsatellite primers, as described above.

Allelic frequencies were tested for the deviations from Hardy–Weinberg equilibrium (HWE) using an exact test with a Markov chain (10000 steps) and 1000 dememorisation steps in Genepop version 4.0.10 [38,39]. Linkage disequilibrium was also tested between all loci using Genepop version 4.0.10 [38,39]. Genetic diversity parameters were computed for each group using GenAlEx v6.4 [40]. The sample sizes in different accessions/regions used in the study were different from each other. Therefore, to compensate for this sampling bias that may lead to inaccurate comparisons of allelic richness between loci, allelic richness (RS) and private allele richness (PR), independent from sample size were computed by a rarefaction method as implemented in HP-RARE version 1.0 [41]. In addition to these genetic diversity parameters, the overall gene diversity (H_T_), the within-population genetic diversity (H_S_), the amount of gene diversity among populations (D_ST_), and the coefficient of genetic differentiation between populations (G_ST_) were calculated with FSTAT version 2.9.3 [42]. To avoid any misinterpretation stemming from sampling bias, D_ST_, H_T_ and G_ST_ values were also calculated independently of sample size, using the same program. Pairwise F_ST_ values between each population were calculated using GenAlEx version 6.4 [40] Population structure was also analyzed using a Bayesian clustering algorithm, as implemented in STRUCTURE version 2.3.3 [43]. Admixture model of ancestry and correlated allele frequency were allowed. The LOCPRIOR model was also applied using population information as a prior, to assist clustering [44]. The length of the burn-in was set to 30,000, and data were collected over 300,000 Markov Chain Monte Carlo (MCMC) replications in each run (K = 1–5). The optimum number of clusters (K), was determined as described by Evanno et al. [45]. Each individual with an ancestry value equal to or larger than 0.7 was assigned to the corresponding cluster, while the individuals with a smaller ancestry value were considered to have mixed ancestry following Coulon et al. [46]. The correspondences of obtained groups were evaluated for taxonomic identity, cultivation status, geographical origin, and climatic zones, as mentioned above. Finally, an Unweighted Pair Group Method with Arithmetic Mean (UPGMA) tree was constructed using Poptree2 [47] based on Nei’s genetic distance (DA) [48] with 10,000 bootstrap iterations.

### Sequence diversity analysis of nuclear EST markers

Varshney et al. [49] had shown that existing barley nuclear expressed sequence tag (EST)-derived DNA markers could be employed in sequence diversity analysis in rye. Four of these markers were tested (S2 Table) and GBS0551, which gave the best results, was selected and used in this study. A total of 61 samples representing four species of *Secale* and five hybrid samples were included in the analysis. The PCR reactions were performed as described by Varshney et al. [49]. The amplified fragments were commercially sequenced at Macrogen Europe and the sequences were edited visually and aligned using Sequencher version 4.5 (Gene Code Corp). However, the discrimination of the alleles of heterozygote samples, especially with multiple differences was not straightforward. Therefore, these sequences were edited by DNAsp version 5.0 [50] using the coalescent-based Bayesian algorithm of PHASE software [51] that resolves haplotype phases and infers haplotypes correctly. A maximum-likelihood (ML) tree was constructed using MEGA 5 [52], and the reliability of the phylogenetic relationships was tested by bootstrapping (1000 replicates).

## Results

### Informativeness of the SSR markers

The number of alleles per locus ranged between 9 and 22 with an average value of 14. Polymorphism information content (PIC) values ranged from 0.605 (REMS1264) to 0.882 (REMS1160), with an average value of 0.718 (Table 1).

**Table 1:** Levels of genetic variability at the seven microsatellite loci.

Locus	N	Na	Ne	PIC	Ho	He
**REMS1187**	659	9	3.09	0.676	0.825	0.676
**REMS1254**	541	17	3.25	0.692	0.698	0.692
**REMS1323**	660	22	3.93	0.745	0.85	0.746
**REMS1264**	644	11	2.54	0.605	0.651	0.606
**REMS1205**	600	11	3.14	0.681	0.733	0.681
**REMS1238**	665	9	3.94	0.745	0.768	0.746
**REMS1160**	576	19	8.48	0.882	0.858	0.882
**Average**	621	14	4.05	0.718	0.769	0.718

N, sample size; N_a_, number of alleles; N_e_, number of effective alleles; PIC, Polymorphism information content; H_o_, observed heterozygosity; He, expected heterozygosity; uHe, unbiased expected heterozygosity.

### Distribution of SSR genetic diversity in different categories

Genetic allelic patterns were calculated for each group, in the three categories created based on taxonomic identity, cultivation status, and climatic conditions of geographical origin of the samples (Table 2). In the taxonomy based groups observed heterozygosity was higher than the expected heterozygosity in all taxa. Expected heterozygosity was the highest in *S*. *strictum* subsp. *strictum* (0.731) and the lowest in *S*. *cereale* subsp. *afghanicum* (0.579), excluding *S*. *cereale* subsp. *dighoricum*, *S*. *strictum* subsp. *irmanuso* and *S*. *sylvestre* that had small sample sizes. The highest and lowest differentiation based on F_ST_ was observed between *S*. *cereale* subsp. *afghanicum* and *S*. *sylvestre* (F_ST_ = 0.181), and *S*. *cereale* and *S*. *vavilovii* (F_ST_ = 0.007), respectively (S3 Table). *S*. *cereale* subsp. *afghanicum* and *S*. *sylvestre* were found to be the most divergent from the rest of the taxa analyzed.

**Table 2:** Mean genetic diversity measures in different panels.

**Taxonomic Identity**	**Group**	**N**	**Na**	**I**	**Ho**	**He**
	*S*. *cereale* subsp. *afghanicum*	3	3.14	1.00	0.67	0.58
	*S*. *cereale* subsp. *ancestrale*	11	5.14	1.34	0.72	0.67
	*S*. *cereale* subsp. *cereale*	533	13.00	1.57	0.77	0.71
	*S*. *cereale* subsp. *dighoricum*	1	1.14	0.40	0.57	0.29
	*S*. *cereale* subsp. *segetale*	36	7.43	1.55	0.80	0.72
	*S*. *strictum* subsp. *anatolicum*	13	5.43	1.32	0.84	0.66
	*S*. *strictum* subsp. *irmanuso*	1	1.29	0.40	0.57	0.29
	*S*. *strictum* subsp. *kuprijanovii*	6	4.14	1.25	0.94	0.67
	*S*. *strictum* subsp. *strictum*	69	8.71	1.63	0.71	0.73
	*S*. *sylvestre*	2	2.29	0.74	0.79	0.48
	*S*. *vavilovii*	46	7.86	1.53	0.81	0.71
**Cultivation Status**	Wild	137	11.29	1.68	0.78	0.74
	Weedy	51	8.43	1.58	0.76	0.73
	Landrace	343	12.86	1.61	0.78	0.72
	Cultivar	190	9.29	1.44	0.76	0.68
**Climate Type**	Tropical monsoon	6	2.57	0.77	0.67	0.46
	Savanna	20	5.00	1.28	0.88	0.67
	Hot semi-arid	11	4.43	1.18	0.75	0.62
	Cold semi-arid	69	9.14	1.58	0.72	0.71
	Hot desert	9	3.86	1.05	0.72	0.57
	Cold desert	15	5.43	1.36	0.73	0.67
	Humid subtropical	64	8.43	1.51	0.78	0.71
	Temperate oceanic	140	9.86	1.55	0.77	0.71
	Hot-summer Mediterranean	88	10.86	1.61	0.76	0.72
	Warm-summer Mediterranean	78	10.00	1.62	0.76	0.73
	Subtropical highland	9	3.71	1.08	0.82	0.60
	Hot-summer humid continental	22	6.43	1.48	0.85	0.71
	Warm-summer humid continental	71	8.14	1.53	0.77	0.71
	Subarctic	19	5.71	1.33	0.81	0.67
	Hot, dry-summer continental	11	5.00	1.19	0.72	0.60
	Warm, dry-summer continental	68	8.71	1.53	0.81	0.70
	Monsoon-influenced humid continental	15	4.86	1.19	0.75	0.61
	Mild tundra	4	3.14	1.00	0.86	0.58

N, sample size; N_a_, number of alleles; I, Information Index H_o_, observed heterozygosity; He, expected heterozygosity; uHe, unbiased expected heterozygosity.

The assessment of cultivation status based genetic diversity in 137 wild, 51 weedy, 343 landrace, and 190 cultivated plants showed that expected heterozygosity was the highest in wild accessions (0.735) and the lowest in cultivated varieties (0.675). Comparison of pairwise F_ST_ values revealed no significant differentiation between different groups.

In terms of the climate subgroups, the highest expected heterozygosity was observed in the Warm-summer Mediterranean subgroup (0.73), and the lowest in Tropical monsoon climate subgroup (0.46) (Table 2). Pairwise F_ST_ comparisons revealed the Tropical monsoon climate subgroup to be the most different from the remaining climate subgroups, with the highest genetic distance when compared to the Mild tundra climate populations (0.19) (S4 Table).

#### STRUCTURE and UPGMA results

**Fig 1:**
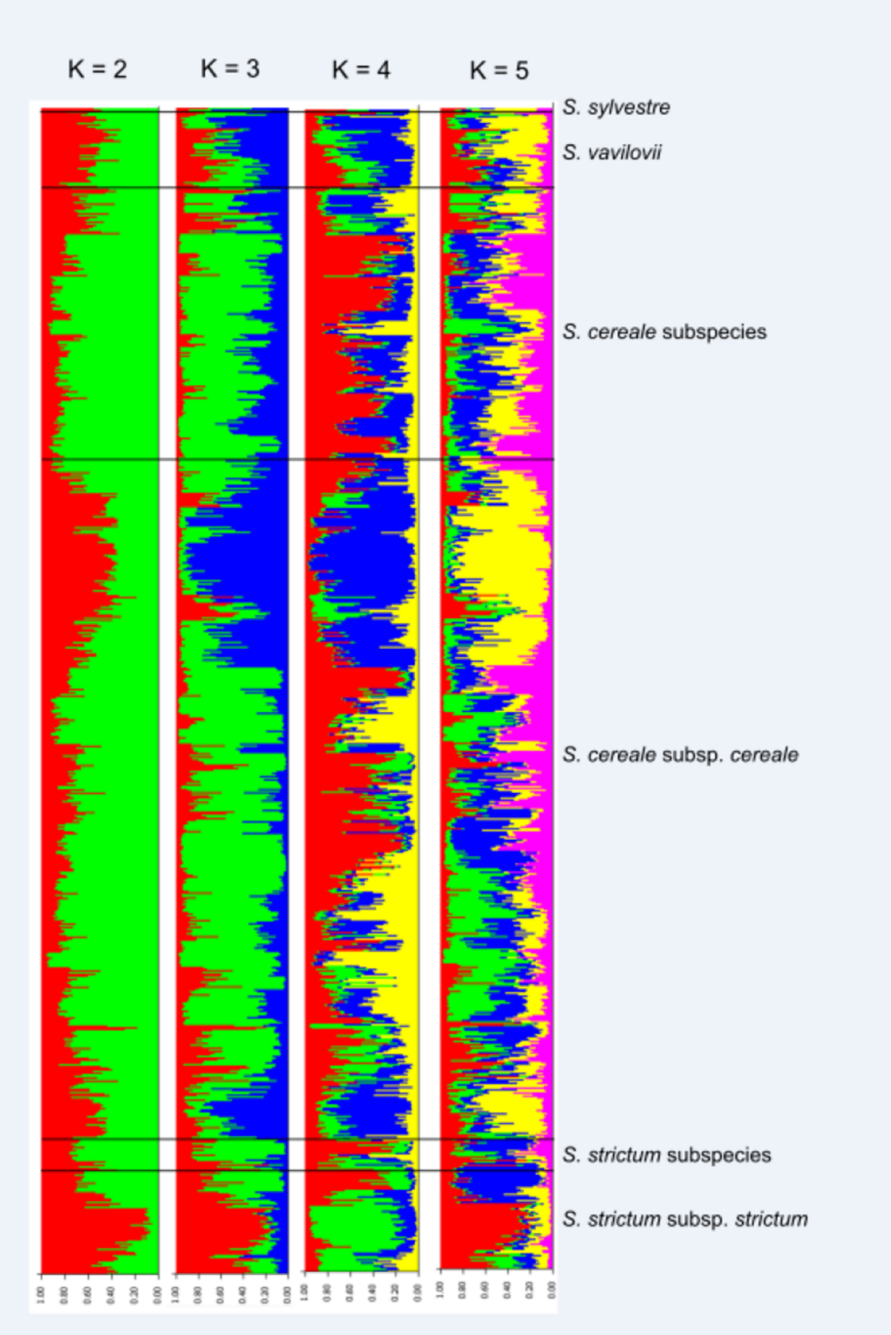
STRUCTURE results at K = 2 to K = 5.

**Fig 2:**
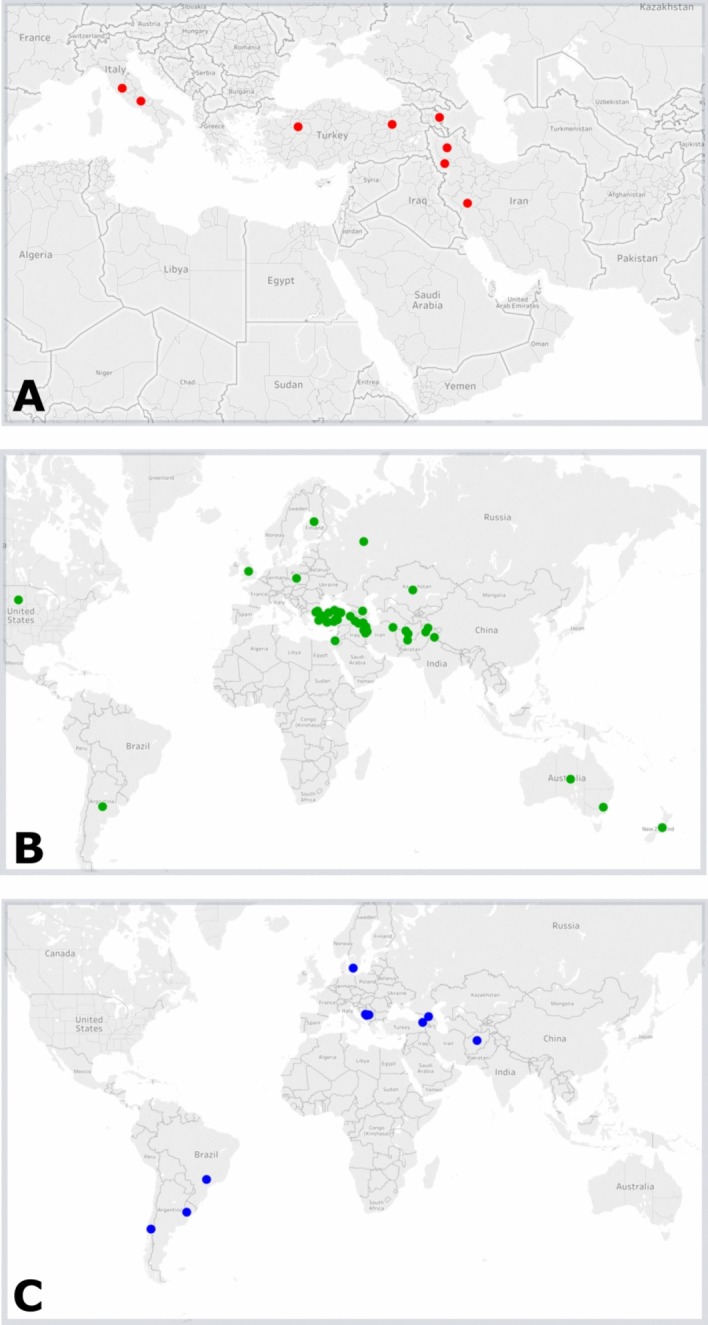
Distribution of samples in different clusters. (A) Distribution of samples in cluster 1. (B) Distribution of samples in cluster 2. (C) Distribution of samples in cluster 3. This figure produced using Tableau Public Software.

The Bayesian clustering analysis based on the distribution of 98 alleles at seven SSR loci among 721 accessions revealed presence of three separate clusters (Fig 1). The primary division at K = 2 was observed mainly between perennial *S*. *strictum* and remaining annual taxa. At K = 3, *S*. *strictum* cluster remained fairly intact, while annual taxa (*S*. *cereale* and *S*. *vavilovi*) grouped within two different clusters. The first cluster contained a total of 25 samples of *S*. *strictum* subsp. *strictum* and one *S*. *strictum* subsp. *anatolicum* sample. Among these, 20 samples originated from Iran, and the remaining samples originated from other parts of the Middle East (Fig 2A). The second cluster included 222 samples of *S*. *cereale* (202 samples of *S*. *cereale* subsp. *cereale*, 13 samples of *S*. *cereale* subsp. *segetale*, six samples of *S*. *cereale* subsp. *ancestrale* and one sample of *S*. *cereale* subsp. *afghanicum*). All of the six *S*. *strictum* subsp. *strictum* samples in cluster 2 originated from Russia. Geographical origins of majority of the *Secale cereale* samples (70.17%) in this cluster corresponded to the Middle East or South-Central Asia (Fig 2B). The third cluster was composed of 66 *S*. *cereale* subsp. *cereale* and three *S*. *vavilovi* samples, most of which (86.4%) originated from out of Asia and the Middle East (Fig 2C). The remaining 408 samples could not be assigned to any of these three clusters, and was considered to have mixed ancestry. Except for the first cluster that consisted of wild *S*. *strictum* samples, a weak correlation between clustering and cultivation status was noted. The structuring exhibited no significant correlation with major agro-climatic zones as described by Kottek et al [31].

**Fig 3:**
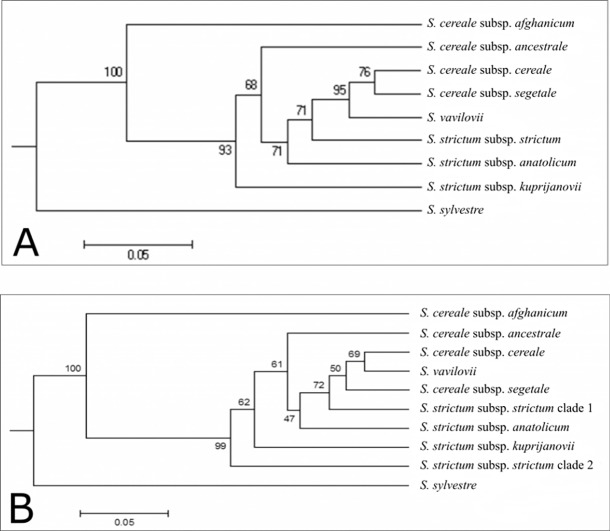
UPGMA dendograms showing the phylogenetic relationship of *Secale* species based on pairwise D_A_. (A) UPGMA dendogram I. (B) UPGMA dendogram II. Bootstrap values are provided on the nodes.

The UPGMA dendogram constructed using subspecies of *S*. *cereale*, subspecies of *S*. *strictum*, *S*. *vavilovii* and *S*. *sylvestre* revealed a clear separation between *S*. *sylvestre* and the rest of genus (Fig 3A). *S*. *cereale* subsp. *afghanicum* separated from the other subspecies of *S*. *cereale* and *S*. *strictum*. *S*. *strictum* subsp. *kuprijanovii* also diverged from the other remaining subspecies at a relatively basal position in the tree topology. *S*. *cereale* subsp. *cereale*, *S*. *cereale* subsp. *segetale*, *S*. *vavilovii*, *S*. *strictum* subsp. *anatolicum* and *S*. *strtictum* subsp. *strictum* constituted a group, while *S*. *cereale* subsp. *ancestrale* remained outside of this cluster. *S*. *cereale* subsp. *cereale* and *S*. *cereale* subsp. *segatale* were more closely related to *S*. *vavilovii*, rather than their conspecifics *S*. *cereale* subsp. *afghanicum* and *S*. *cereale* subsp. *ancestrale*.

As the STRUCTURE analysis revealed a clear separation between *S*. *strictum* subsp. *strictum* samples originating from Iran, as a next step, these populations were grouped separately (*S*. *strictum* subsp. *strictum* clade 1). The remaining *S*. *strictum* populations were also grouped together (*S*. *strictum* subsp. *strictum* clade 2), and the dendogram was rebuilt using these separated groups (Fig 3B). Branching off of *S*. *strictum* subsp. *strictum* clade 2 with a high bootstrap value (99%) revealed its significant divergence. Except for this difference, both trees reflected nearly identical topologies.

### Nuclear sequence diversity of the genus *Secale*

**Fig 4:**
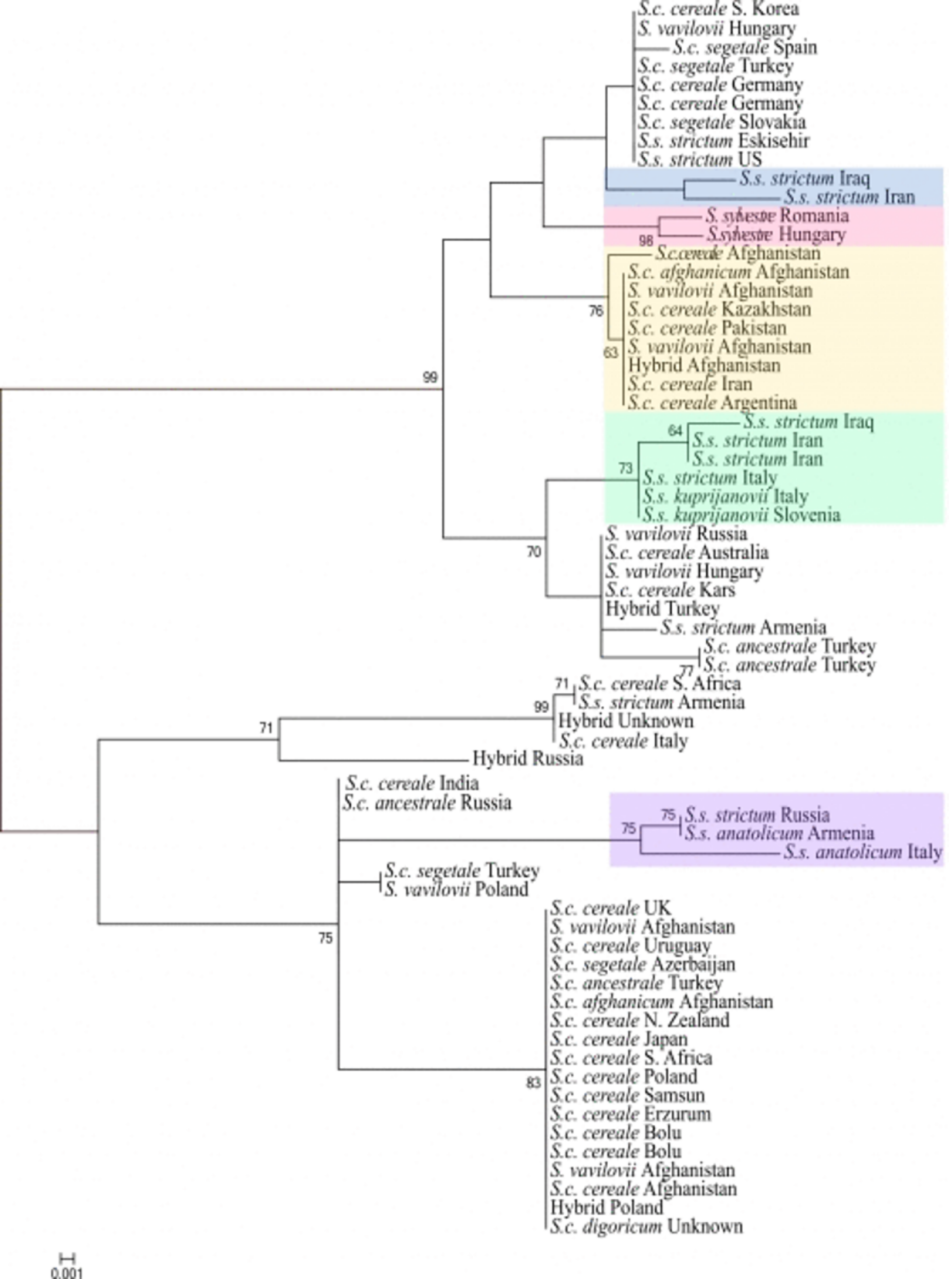
Maximum likelihood phylogenetic tree based on the nuclear GBS0551 region.

The general topology of the maximum-likelihood (ML) tree constructed using a 667 bp fragment of nuclear sequences in 61 samples (GenBank accession numbers: MH421898-MH421958), representing four species in the genus *Secale*, and five hybrid samples showed that there were two main lineages (Fig 4). However, these groups did not correspond to taxonomic or spatial delimitations. *S*. *vavilovii* accessions were dispersed within *S*. *cereale* subspecies in both groups. The two *S*. *sylvestre* samples clustered together in a subgroup, rather than forming a separate linage. *S*. *strictum* subspecies clustered together forming two and one subgroups within the first and second lineage, respectively. Furthermore, a separated subgroup was recovered within the first linage containing *S*. *cereale* and *S*. *vavilovii* samples from South and Central Asia, except for a *S*. *cereale* subsp. *cereale* sample originating from Argentina. In the second group, no clear relationships were recognized between the genealogy of *Secale* species and their geographic origin.

### Microsatellite based genetic diversity and population structure of the cultivated rye (*Secale cereale* subsp. *cereale*)

In the genetic diversity analysis conducted exclusively in landrace and cultivated rye samples, the total number of alleles and private alleles were 96 and 26, respectively (Table 3). The highest mean corrected allelic richness was found in the Middle East populations (3.68), and lowest in the Africa population (2.76). Private allelic richness independent of sample size was also the highest in the Middle Eastern populations (0.35), and lowest in South America and South-Central Asia populations (0.14). The observed heterozygosity levels were higher than expected heterozygosity levels in all geographical regions. The mean expected heterozygosity was highest in the Middle East gene pool (0.72), and lowest in the African gene pool (0.51). Shannon’s information index (I) was again highest in the Middle Eastern populations (1.62), and lowest in African population (0.85). The corrected total genetic diversity (H_T_′) in cereal rye showed variations from region to region, and found to be highest in the Middle East (0.74) and Caucasus (0.71), and lowest in East Asia (0.62). Intra-population genetic diversity (H_S_), the measure of average differences within populations was found to be the highest in the Middle East and Caucasus populations (0.70), and lowest in North American populations (0.59).

**Table 3:** Levels of genetic variability at seven microsatellite loci for cultivated rye.

Region	N	NA	RS	Ne	I	Ho	PA	PR	He	Hs	H_T_	H_T_′	D_ST_	D_ST_′	G_ST_
**Africa**	6	3.14	2.76	2.17	0.85	0.65	0.00	0.18	0,51	Nd	Nd	Nd	Nd	Nd	Nd
**Australia**	32	5.86	3.13	3.38	1.25	0.74	0.29	0.20	0,63	0.64	0.65	0.66	0.01	0.01	0.01
**Balkans**	62	7.14	3.44	3.69	1.43	0.80	0.29	0.21	0,68	0.63	0.70	0.71	0.07	0.08	0.10
**Caucasus**	14	5.29	3.53	3.24	1.34	0.80	0.00	0.34	0,68	0.70	0.71	0.73	0.02	0.03	0.02
**East Asia**	31	5.71	3.19	2.95	1.22	0.73	0.00	0.21	0,58	0.62	0.62	0.62	0.00	0.01	0.01
**Europe**	36	6.71	3.46	3.58	1.42	0.80	0.00	0.21	0,68	0.65	0.68	0.69	0.03	0.04	0.05
**Middle East**	240	12.43	3.68	4.06	1.62	0.77	2.86	0.35	0,72	0.70	0.74	0.74	0.04	0.04	0.06
**N. America**	22	5.71	3.32	3.20	1.30	0.77	0.14	0.17	0,65	0.59	0.64	0.67	0.05	0.08	0.08
**South America**	43	6.00	3.42	3.29	1.36	0.82	0.00	0.14	0,67	0.65	0.68	0.69	0.03	0.04	0.05
**South and** **Central Asia**	47	6.43	3.20	3.27	1.29	0.74	0.14	0.14	0,63	0.64	0.66	0.66	0.01	0.02	0.02

N, sample size; N_A_, number of alleles; R_S_, allelic richness; N_E_, number of effective alleles; P_R_, number of private alleles independent of sample size; I, Shannon’s information index; H_O_, observed heterozygosity; H_E_, expected heterozygosity; P_R_, private allelic richness; H_S_, the within population genetic diversity; H_T_’_,_ the total genetic diversity independent of sample size; D_ST_′ among-populations genetic diversity independent of sample size; G_ST_′ the coefficient of genetic differentiation independent of sample size.

The comparison of coefficient of among-populations genetic diversity independent of sample sizes (D_ST_′), and coefficient of gene differentiation independent of sample sizes (G_ST_′) as the measures of genetic differentiation between populations in each region was found to be highest in North America (D_ST_′ = 0.08, G_ST_′ = 0.12) and the Balkans (D_ST_′ = 0.08, G_ST_′ = 0.11), and lowest in East Asia (D_ST_′ = 0.01, G_ST_′ = 0.01) (Table 3). Furthermore, comparison of pairwise F_ST_ differentiation [53, 54] showed the African gene pool to be the most different from remaining gene pools, having the highest genetic distance when compared to the South-Central Asian populations (F_ST_ = 0.96) (S5 Table). The genetic differentiation among other gene pools was insignificant.

STRUCTURE analysis showed the presence of two separate clusters, with the first one composed of 333 samples, 72.02% of which were landraces that originated from the Middle East, and South and Central Asia. Except for two samples, all of the Australian cultivars clustered in this group. The second cluster was composed of 136 samples, mainly originating from Europe, Balkans and South America. The proportion of Middle Eastern and south Central Asian samples in this group was only 6.25%.

**Fig 5:**
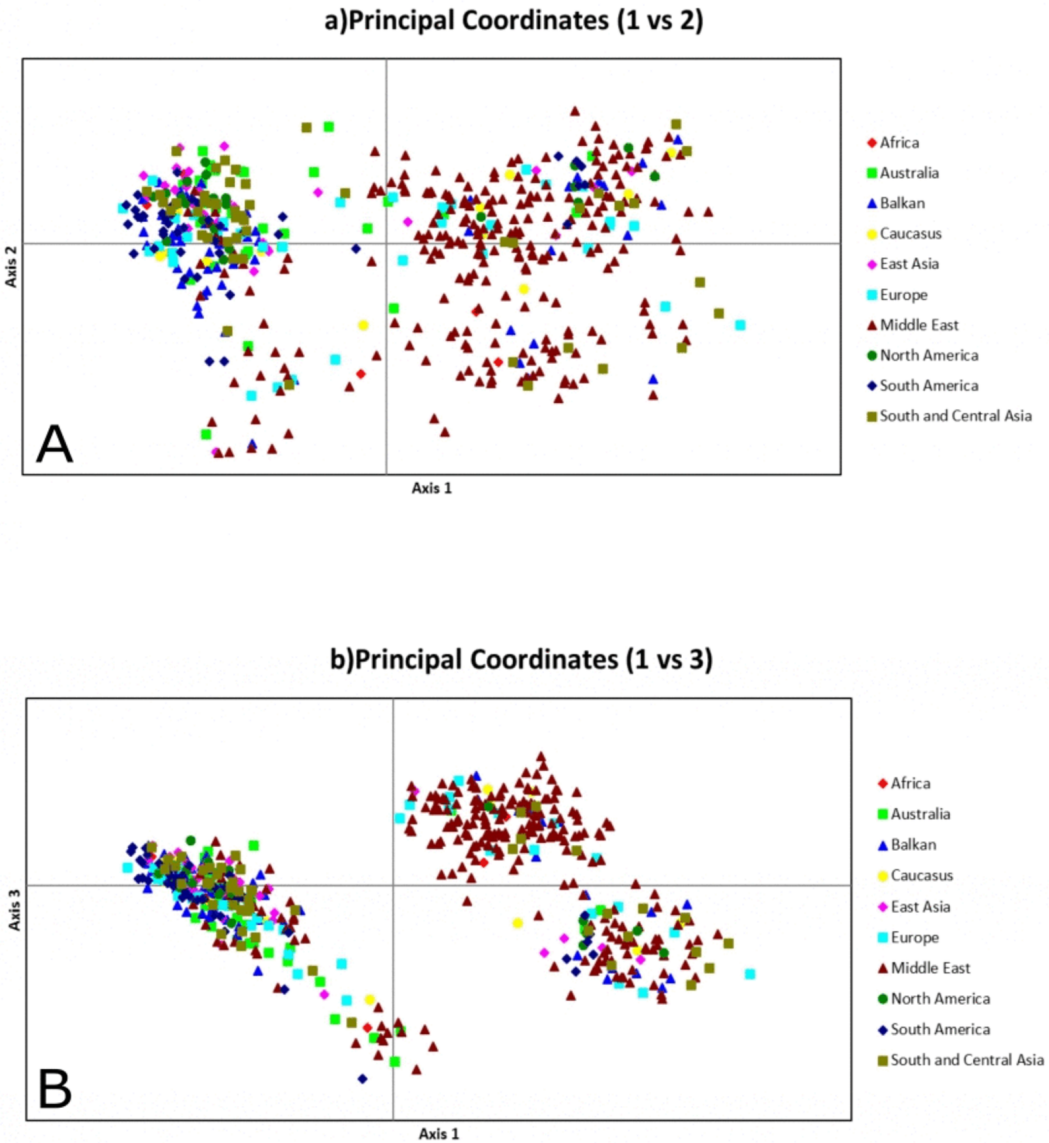
PCA analysis of *S*. *cereale* subsp. *cereale* samples with different geographical origins. (A) 1^st^ and 2^nd^ components. (B) 1^st^ and 3^rd^ components.

In the PCA analysis conducted to explore pattern of relationship between cultivated rye populations from different geographical regions with the microsatellite data, the first, second and third components explained 45.44%, 23.37% and 12.54% of the variance, respectively. First and second components of the PCA analysis revealed two clusters (Fig 5A), while three distinct clusters were observed based on the first and third components (Fig 5B). The first cluster was dominated by samples from the Middle East, whereas the other clusters contained samples from diverse geographical areas. PCA clustering did not reflect cultivation status of the samples.

## Discussion

### Distribution of SSR diversity in *Secale*

In this study, genetic diversity within *Secale* was evaluated using seven SSR markers. A world-wide collection of 721 samples belonging to 11 taxonomic units included wild, weedy, landrace and cultivated materials from diverse climatic zones. All of the SSR markers employed in the study had (PIC) values higher than 0.6 and are considered to be highly informative. The genetic diversity of*Secale* at a global scale was relatively high compared to other crops like sorghum [55] and maize [56], which can be attributable to the outcrossing nature of many species in the genus *Secale*, and its wind-pollinated reproduction.

In our study, relative genetic diversity co-varied with the cultivation status: the highest diversity was observed in wild accessions, followed by weedy and landrace accessions, and lowest in cultivated varieties. Furthermore, wild and landrace populations had private alleles which were not detected in the cultivated gene pools, indicating that these forms offer a richer source of alleles, and high potential for crop improvement. High genetic diversity and presence of private or rare alleles in wild and weedy forms can be explained by the lack of a domestication bottleneck, see below.

### Genetic clustering

Existence of three distinct clusters in the STRUCTURE analyses of the whole genus indicated the presence of three different gene pools: (1) perennial *S*. *strictum* subsp. *strictum*, (2) annual taxa that originated from Asia (Middle East and South-Central Asia), (3) annual taxa that originated from outside of Asia (mainly Europe). The clear separation between perennial *S*. *strictum* and the annual form has been shown previously [11,57,58] and can be explained by restricted gene-flow between annual and perennial taxa, possibly due to the differences in life-history traits such as timing of reproduction.

Further separation of annual taxa was based on geographic origin, rather than taxonomic identity. This was also supported by maximum likelihood tree constructed using nuclear sequences, where all of the *S*. *cereale* subspecies and *S*. *vavilovii* were grouped together. Recently, Hagenblad et al. [11] showed that there was no clear taxonomic structuring among annual forms of the genus. Previous studies have also shown lack of morphological [59] and molecular [8,9,10,57] differences between annual forms (*S*. *vavilovii*, *S*. *cereale* subsp. *ancestrale*, *S*. *cereale* subsp. *afghanicum*, and *S*. *cereale* subsp. *segetale*) belonging to different taxa. Genetic similarity between annual wild and weedy forms and cultivated subspecies *S*. *cereale* subsp. *cereale* supports the hypothesis that *S*. *cereale* is of relatively recent origin, dating back to only a few centuries ago [60]. It is likely that there was insufficient time for the evolution of isolation mechanisms or barriers between cultivated rye, and its wild and weedy relatives, and hence the lack of structuring among annual taxa can be explained by introgression between sympatric populations of cultivated rye, and wild and weedy forms. As a result, morphological differences between the subspecies cannot be explained by genetic differentiation indicating the lack of nonexistence of the taxonomic boundaries at subspecies level. Interbreeding between different taxa, except for *S*. *sylvestre* and subsequent formation of hybrids with high pollen and seed fertility is very common in the *Secale* genus [6,20,61,62].

The further structuring of annual taxa based on geographic origins of samples suggests that each of the two annual clusters detected in the study originated from two distinct gene pools. Subsequently, the two distinct lineages retrieved in this study were initially separated, probably due to restriction of gene flow because of geographical isolation. Consistent with our findings, Hagenblad et al. [11] also showed that geographic clustering was evident among annual taxa, which is reflected by a separation between Asian and European accessions. Furthermore, Bolibok-Bragoszewska et al. [63] noted divergence of the Near Eastern and European accessions. On the other hand, some other studies reported that genetic structuring among different taxa corresponded to cultivation status [58,64–66], and no geographical structuring was found in these studies. These conflicting results might be stemming from low discriminatory power of markers used in these studies. It should be noted that despite the existence of the clear geographical structuring among annual taxa mentioned above, no significant correlation with major agro-climatic zones was detected. Similarly, Hagenblad et al. [11] also reported a limited correlation between genetic structuring and agro-climatic conditions of the sampling localities in geographically structured rye populations. This can be explained by the observed structure stemming from geographical proximity and related pollen dispersal, rather than ecological and climatic adaptations.

The nominotypic *S*. *strictum* subsp. *strictum* has been previously shown to be significantly different from its subspecies [8,9,10, 67], indicating that it has been evolving independently of other *S*. *strictum* subspecies [13]. In our study *S*. *strictum* subsp. *strictum* samples from northwest and west of Iran were divergent from the rest of the *S*. *strictum* accessions. In the UPGMA dendogram, the ancestral position of this group was observed, when compared to the rest of *S*. *strictum* subsp. *strictum* samples. The same dendogram also revealed that rest of the *S*. *strictum* subsp. *strictum* samples (*i*.*e*. other than the Iranian clade) originating from diverse areas, were genetically more similar to *S*. *cereale* accessions. This is compatible with the hypothesis that cultivated rye evolved from *S*. *strictum* [17,20,60]. In addition, in the dendogram constructed based on microsatellite data, *S*. *strictum* subsp. *anatolicum* and *S*. *strictum* subsp. *strictum* accessions originating from out of Iran were found to be closer to S. *cereale* subspecies compared to *S*. *strictum* subsp. *kuprijanovii* samples, and *S*. *strictum* subsp. *strictum* accessions originating from Iran. This suggests existence of gene flow between *S*. *cereale* subspecies and *S*. *strictum* subspecies originating from outside of Iran.

It was also interesting that *S*. *strictum* subsp. *strictum* samples that originated from Iran were basal to the clade that included the rest of *S*. *strictum* subspecies and all of the annual taxa, except for *S*. *cereale* subsp. *afghanicum* and *S*. *sylvestre*. This observation is also consistent with previous studies that show *S*. *strictum* being the most ancestral species, which the rest of the taxa have originated from[4,6,20, 68,69]. This finding also underpins the hypothesis that Northeastern Turkey and the adjacent area including Armenia and northwestern Iran could be the center of origin for the genus [5,18].

Taxonomical position of *S*. *vavilovii* has also been a point of discussion: in some studies, *S*. *vavilovii* was considered to be a distinct species close to *S*. *cereale* [6,8,15,57,58, 70], while some other researchers postulated that *S*. *vavilovii* should be classified as a subspecies of *S*. *cereale* [7,9–11,13,67]. In our study, SSR and nuclear sequence diversity analysis did not reveal significant differentiation between *S*. *cereale* and *S*. *vavilovii*. Therefore, it is concluded that *S*. *vavilovii* should be considered as a synonym and a subspecies of *S*. *cereale*.

Our study affirmed that *S*. *sylvestre* is genetically the most divergent species, which is consistent with the general agreement that *S*. *sylvestre* is the first species that diverged from *S*. *strictum* during the Pliocene, and is morphologically and genetically the most distinct species [12,13,71].

### Genetic diversity and structuring of cultivated rye

#### Genetic diversity

Cultivated rye is a wind pollinated allogamous species with a highly developed self-incompatibility system. As a result, high genetic diversity has been previously noted not only between different accessions [24,72,73], but also within the same cultivar [9,10,65]. Consistently, our study showed high degrees of genetic diversity in cultivated rye from all over the world. Moreover, due to its high tolerance of different environmental conditions, rye has a global geographic distribution which may also have contributed to its high levels of genetic diversity.

In the scope of the present study, genetic diversity levels of different gene pools were compared. Our results showed that landraces are genetically more diverse, when compared to cultivars. Similar results were previously reported in other studies on rye [23,61]. It is well established that current breeding practices narrows genepool and leads to reduction of genetic diversity [74]. Such reduction in genetic diversity results in loss of many important alleles, and this may have significant negative effects on adaptive capacity of plants. On the other hand, landraces are cultivated by traditional agricultural practices through many generations of selection, and they have become locally adapted to various environments by accumulating new alleles [75]. Therefore, compared to cultivars, the genetic diversity of landraces is high. Our findings highlighted that landraces should be regarded as a source of genetic variation, and should be integrated to rye breeding programs to compensate genetic diversity lost during modern breeding processes.

Second, we analyzed the distribution of genetic diversity in different geographic regions. Genetic diversity of cultivated rye was affected by geographic origins of the samples and found to be higher in the Middle East region (Turkey, Iran and Israel) compared to other regions. Although sample size of the region is larger than the others, to avoid any bias due to sample size, corrected genetic diversity measures (independent of sample size) were also used. The degree of genetic diversity was found to be highest in the Middle East for the corrected parameters, as well. Therefore the obtained results likely reflect real genetic diversity patterns of the region, rather than being a sampling artifact. Vavilov [17] proposed that genetic diversity of crop species on interspecific and intraspecific level is not evenly distributed: the genetic diversity in the center of origins is higher. Based on this assumption, our results indicate that the most likely center of origin for the genus is the Middle East or Caucasus. This is consistent with the idea that all *Secale* taxa have originated somewhere in the Middle East or South-Central Asia [5,18] that also covers the geographical area known as “Fertile Crescent”, the center of origin for many crop species like wheat, barley, pulses, pea and flaxes [18]. Taking into account that many wild and weedy forms of the genus *Secale* are found in the area between northeastern Turkey and northwestern Iran, gene flow between wild forms and cultivated forms by introgression is quite possible, resulting in an increase in genetic diversity. High genetic diversity observed in the region can also be explained by most of the populations in this region being landraces, rather than genetically more-or-less uniform cultivars.

Genetic diversity of the accessions originating from Africa and East Asia was comparably low, probably due to a potential genetic bottleneck during introduction of cultivated rye to these regions. Besides, in comparison to South American and European samples, genetic diversity was lower in North America that contains populations from Mexico, USA and Canada. This probably stems from extensive use of genetically uniform cultivars in these regions. On the other hand, genetic diversity of the Balkan group (that contained samples from European part of Turkey (Thrace), Montenegro, Serbia, Macedonia, Yugoslavia, and Bosnia and Herzegovina) and the Caucasus gene pool (that consisted of two accessions from Georgia and East Azerbaijan) was high. Finally, the European gene pool sampled in this study contained accessions from a wide geographical range containing Germany, Switzerland, UK, Poland and Sweden, and their genetic diversity levels were found to be moderately high. Although agricultural systems of many countries in Europe favor the genetically uniform cultivars [76], the relatively high levels of genetic diversity observed could be explained by European cultivars having been developed using different genepools.

#### Origins of the ryes from different continents

The separation of genotypes originating from Asia (Middle Eastern, and south and central Asian) and from out of Asia (mainly Europe, Balkans and South America) was consistent with previous studies reporting a clear separation between the Middle East and European genepools [11,23,63].

Based on our findings it can be speculated that each of the two clusters obtained in the study originated from two distinct gene pools. The two main distinctive lineages retrieved in this study were initially separated probably due to restriction of gene flow because of geographical separation. Considering that in crop plants geographical distribution patterns usually reflect prevailing human mediated selection pressures in a particular environment [77], another explanation for this separation could be the cultivated rye having been introduced into new geographical ranges in which climatic and environmental conditions are quite different compared to those in the center of origin. This was possibly followed by anthropogenic selection of adaptable phenotypes to the conditions in those regions, leading to adaptive divergence. The Middle Eastern samples were observed in all three clusters, indicating their potential ancestral position, and supporting the conclusion -based on genetic diversity levels above- that the Middle Eastern populations are the likely progenitors of cultivated rye, and they recently expanded globally due to human mediated distribution and long-distance gene flow. Similarly, Einkorn wheat, emmer wheat, barley and lentil [78] were domesticated in the Middle East, more specifically in the Fertile Crescent and subsequently were radiated to Europe [79] and the rest of the world.

In the context of the study, the origin of the samples collected from outside of Asia and Europe was also investigated. Samples from South America grouped together with European samples into second cluster. This is consistent with the idea that many crop plants dispersed to South America from Europe, after the voyages of Columbus [80]. On the other hand, samples from Australia and North America grouped into the first cluster, indicating that cultivated rye was possibly introduced into these areas from the Middle East or South-Central Asia.

Furthermore, genetic differentiation among geographical regions revealed a significant differentiation between the African gene pool and the remaining gene pools. Considering climatic conditions of the region being relatively unique and that the region is physically separated from remaining gene pools by geographical barriers, it can be concluded that rye became locally adapted to this continent and remained separated. This is consistent with the idea that *S*. *cereale* subsp. *cereale* evolved as an isolated population in Africa [5]. Similarly, based on AFLP data, Chikmawati [73] previously reported that African populations of cultivated rye were genetically more distant when compared to other populations.

## Conclusion

The global scale analysis of genetic diversity and phylogenetic relationships of *Secale* genus show a clear separation between perennial *S*. *strictum* subspecies and annual taxa. Further separation of annual taxa belonging to different species or subspecies into two groups was based on geographical origin, rather than taxonomic identity. Separation of the Middle Eastern and South Central Asian accessions from remaining accessions confirmed the previous findings revealing partitioning between Asian and European accessions, and the existence of two different genepools. The lack of any structuring within different species or subspecies belonging to annual taxa can be explained by recent separation of the species or subspecies, insufficient time having passed for the evolution of isolation mechanisms, and consequent continuation of gene flow even between species. In addition, the lack of a clear genetic separation between *S*. *cereale* and *S*. *vavilovii* led us to conclude that *S*. *vavilovii*, rather than being a distinct species, should be classified as a subspecies of *S*. *cereale*. The phylogenetic relationships of different species in the genus should be investigated in greater detail using high resolution molecular markers, such as RAD-seq, as well.

The evaluation of genetic diversity of cultivated rye populations led us to conclude that high levels of genetic variation exist in cultivated rye. The highest allelic variation and genetic diversity was found in the Middle Eastern landrace populations. This finding supports the idea that the area could be the center of origin for the genus. Nearly all of the populations examined in Near East are locally adapted landraces that have not been exposed to intense artificial selection pressures. Therefore, in contrast to modern crop varieties that have undergone genetic bottlenecks associated with the process of domestication, resulting in a decrease in genetic diversity, landraces constitute a large pool of genetic variation and contain many interesting traits, like strong tolerance to abiotic and biotic stress [81]. Considering that high genetic diversity in crop plant populations is directly related to adaptive potential of those populations to changing environmental conditions, landraces should be regarded as genetic resources reservoirs for new niches and future breeding programs. From a conservation point of view, the results obtained from the study suggest that an immediate action plan is required for in-situ conservation of the ancestral and highly diverse Middle Eastern landrace populations.

## Supporting information

S1 Table Plant populations used in the study. Am, Tropical monsoon climate; Aw, Savanna; BSh, Hot semi-arid; BSk, Cold semi-arid; BWh, Hot desert; BWk, Cold desert; Cfa, Humid subtropical climate; Cfb,Temperate oceanic climate; Csa, Hot-summer Mediterranean climate; Csb, Warm-summer Mediterranean climate; Cwb, Subtropical highland climate; Dfa, Hot-summer humid continental climate; Dfb, Warm-summer humid continental climate; Dfc, Subarctic climate; Dsa Hot, dry-summer continental climate; Dsb, Warm, dry-summer continental climate; Dwa, Monsoon-influenced hot-summer humid continental climate; ET, Mild tundra climate.
(DOCX)

S2 Table Molecular markers used in the study. Chr, Chromosome number; Temp, Annealing Temperature (°C); ref., Reference.
(DOCX)

S3 Table Comparison of pairwise F_ST_ genetic distances between subspecies. S. c. afg., *S*. *cereale* subsp. *afghanicum*; S. c. anc., *S*. *cereale* subsp. *ancestrale*; S. c. cereale, *S*. *cereale* subsp. *cereale*; S. c. dighor., *S*. *cereale* subsp. *dighoricum*; S. c. segetale, *S*. *cereale* subsp. *segetale;* S. s. anatol, *S*. *strictum* subsp. *anatolicum*; S. s. irm. *S*. *strictum* subsp. *irmanuso*; S. s. kupr., *S*. *strictum* subsp. *kuprijanovii*; S. s. strictum, *S*. *strictum* subsp. *strictum*; S. sylv., *S*. *sylvestre*; S. vav., *S*. *vavilovii*. Significant *P* values are indicated in bold.
(DOCX)

S4 Table Comparison of pairwise F_ST_ genetic distances between climate zone groups. Am, Tropical monsoon climate; Aw, Savanna; BSh, Hot semi-arid; BSk, Cold semi-arid; BWh, Hot desert; BWk, Cold desert; Cfa, Humid subtropical climate; Cfb,Temperate oceanic climate; Csa, Hot-summer Mediterranean climate; Csb, Warm-summer Mediterranean climate; Cwb, Subtropical highland climate; Dfa, Hot-summer humid continental climate; Dfb, Warm-summer humid continental climate; Dfc, Subarctic climate; Dsa Hot, dry-summer continental climate; Dsb, Warm, dry-summer continental climate; Dwa, Monsoon-influenced hot-summer humid continental climate; ET, Mild tundra climate.
Significant *P* values are indicated in bold.
(DOCX)

S5 Table The pairwise F_ST_ values of cultivated rye each geographical region calculated using seven microsatellite markers. Significant *P* values are indicated in bold.
(DOCX)
