# Supplementary material for: Phylogeny and genetic structure in the genus Secale
Source: PLoS One. 2018 Jul 19;13(7):e0200825. doi: 10.1371/journal.pone.0200825 (PMC6053196; doi:10.1371/journal.pone.0200825)
Supplement: S1 Table — Am, Tropical monsoon climate; Aw, Savanna; BSh, Hot semi-arid; BSk, Cold semi-arid; BWh, Hot desert; BWk, Cold desert; Cfa, Humid subtropical climate; Cfb,Temperate oceanic climate; Csa, Hot-summer Mediterranean climate; Csb, Warm-summer Mediterranean climate; Cwb, Subtropical highland climate; Dfa, Hot-summer humid continental climate; Dfb, Warm-summer humid continental climate; Dfc, Subarctic climate; Dsa Hot, dry-summer continental climate; Dsb, Warm, dry-summer continental climate; Dwa, Monsoon-influenced hot-summer humid continental climate; ET, Mild tundra climate. (DOCX) [file pone.0200825.s001.docx]

**S1 Table. Plant populations used in the study.**  Am, Tropical monsoon climate; Aw, Savanna; BSh , Hot semi-arid; BSk, Cold semi-arid; BWh, Hot desert; BWk, Cold desert; Cfa, Humid subtropical climate; Cfb,Temperate oceanic climate; Csa, Hot-summer Mediterranean climate; Csb, Warm-summer Mediterranean climate; Cwb, Subtropical highland climate; Dfa, Hot-summer humid continental climate; Dfb, Warm-summer humid continental climate; Dfc, Subarctic climate; Dsa Hot, dry-summer continental climate; Dsb, Warm, dry-summer continental climate; Dwa, Monsoon-influenced hot-summer humid continental climate; ET, Mild tundra climate.

| **Taxon** | **Accession no** | **No of  Samp.** | **Climate code** | **Origin** | **Cultivation Status** | **Lat.** | **Long.** | **Source** |
| --- | --- | --- | --- | --- | --- | --- | --- | --- |
| *S. cereale afghanicum* | R1038 | 1 | BWk | Afghanistan | Weedy | 36 | 65 | IPK |
| *S. cereale afghanicum* | R567 | 1 | BWk | Afghanistan | Weedy | 35 | 65 | IPK |
| *S. cereale afghanicum* | R569 | 1 | BWk | Afghanistan | Weedy | 36 | 65 | IPK |
| *S. cereale dighoricum* | R250 | 1 | Unknown | Unknown | Weedy |  |  | IPK |
| *S. cereale segetale* | PI326284 | 12 | Dfb | Azerbaijan | Weedy | 42 | 47 | USDA |
| *S. cereale segetale* | PI326286 | 12 | BSk | Kazakhstan | Weedy | 48 | 68 | USDA |
| *S. cereale segetale* | PI618671 | 2 | Csb | Turkey | Weedy | 39 | 35 | USDA |
| *S. cereale segetale* | PI618673 | 6 | BSk | Turkey | Weedy | 38 | 33 | USDA |
| *S. cereale segetale* | R279 | 1 | Csb | Turkey | Weedy | 40 | 36 | IPK |
| *S. cereale segetale* | R607 | 1 | Dfb | Slovakia | Weedy | 49 | 20 | IPK |
| *S. cereale segetale* | R61 | 1 | Dfa | Russian Federation | Weedy | 48 | 46 | IPK |
| *S. cereale segetale* | R788 | 1 | BSk | Spain | Weedy | 40 | -4 | IPK |
| *S. cereale ancestrale* | PI618666 | 7 | Csb | Turkey | Weedy | 39 | 35 | USDA |
| *S. cereale ancestrale* | R1054 | 1 | BWk | Turkmenistan | Weedy | 40 | 60 | IPK |
| *S. cereale ancestrale* | R29 | 1 | Dfa | Russian Federation | Weedy | 48 | 46 | IPK |
| *S. cereale ancestrale* | R62 | 1 | Csb | Turkey | Weedy | 40 | 36 | IPK |
| *S. cereale ancestrale* | R767 | 1 | Csa | Turkey | Weedy | 38 | 36 | IPK |
| *S. cereale cereale* | CIse 108 | 12 | Dfb | Japan | Cultivar | 36 | 138 | USDA |
| *S. cereale cereale* | CIse 110 | 15 | Dwa | South Korea | Cultivar | 37 | 128 | USDA |
| *S. cereale cereale* | CIse 12 | 9 | Dfa | US | Cultivar | 45 | -100 | USDA |
| *S. cereale cereale* | CIse 37 | 10 | Dsb | Afghanistan | Landrace | 34 | 66 | USDA |
| *S. cereale cereale* | CIse 79 | 9 | BWh | Australia | Cultivar | -25 | 135 | USDA |
| *S. cereale cereale* | PI168130 | 5 | Csa | Turkey | Landrace | 37 | 31 | USDA |
| *S. cereale cereale* | PI168136 | 6 | Csa | Turkey | Landrace | 38 | 27 | USDA |
| *S. cereale cereale* | PI168168 | 7 | Csb | Turkey | Landrace | 40 | 29 | USDA |
| *S. cereale cereale* | PI168176 | 8 | Csb | Turkey | Landrace | 38 | 32 | USDA |
| *S. cereale cereale* | PI168181 | 3 | Cfb | Turkey | Landrace | 41 | 36 | USDA |
| *S. cereale cereale* | PI168194 | 1 | Csb | Turkey | Landrace | 40 | 35 | USDA |
| *S. cereale cereale* | PI168195 | 7 | Csa | Turkey | Landrace | 42 | 28 | USDA |
| *S. cereale cereale* | PI168196 | 4 | Csa | Turkey | Landrace | 41 | 27 | USDA |
| *S. cereale cereale* | PI168199 | 8 | Csb | Turkey | Landrace | 38 | 31 | USDA |
| *S. cereale cereale* | PI168205 | 3 | Csb | Turkey | Landrace | 40 | 34 | USDA |
| *S. cereale cereale* | PI168209 | 6 | Csb | Turkey | Landrace | 39 | 30 | USDA |
| *S. cereale cereale* | PI168211 | 3 | Csb | Turkey | Landrace | 39 | 31 | USDA |
| *S. cereale cereale* | PI168213 | 4 | Csb | Turkey | Landrace | 39 | 34 | USDA |
| *S. cereale cereale* | PI168218 | 9 | Dsb | Turkey | Landrace | 39 | 36 | USDA |
| *S. cereale cereale* | PI168220 | 14 | Csb | Turkey | Landrace | 38 | 34 | USDA |
| *S. cereale cereale* | PI173587 | 1 | Csb | Turkey | Landrace | 41 | 36 | USDA |
| *S. cereale cereale* | PI173589 | 11 | Cfa | Turkey | Landrace | 41 | 37 | USDA |
| *S. cereale cereale* | PI205221 | 1 | Csb | Turkey | Landrace | 40 | 33 | USDA |
| *S. cereale cereale* | PI218110 | 4 | Cfa | Pakistan | Landrace | 35 | 73 | USDA |
| *S. cereale cereale* | PI220118 | 3 | BSk | Afghanistan | Landrace | 32 | 66 | USDA |
| *S. cereale cereale* | PI227870 | 4 | Csa | Iran | Landrace | 32 | 51 | USDA |
| *S. cereale cereale* | PI228360 | 1 | Csa | Iran | Landrace | 35 | 47 | USDA |
| *S. cereale cereale* | PI234655 | 9 | BSk | Kazakhstan | Cultivar | 48 | 68 | USDA |
| *S. cereale cereale* | PI240675 | 17 | Cfa | Uruguay | Cultivar | -34 | -58 | USDA |
| *S. cereale cereale* | PI243741 | 1 | Dsb | Iran | Landrace | 38 | 45 | USDA |
| *S. cereale cereale* | PI250745 | 2 | Dsa | Iran | Landrace | 38 | 47 | USDA |
| *S. cereale cereale* | PI252002 | 8 | Csa | Turkey | Landrace | 39 | 32 | USDA |
| *S. cereale cereale* | PI261400 | 3 | Dfc | Canada | Cultivar | 50 | -86 | USDA |
| *S. cereale cereale* | PI268281 | 9 | Csa | Iran | Landrace | 34 | 48 | USDA |
| *S. cereale cereale* | PI289814 | 11 | BSk | Iran | Landrace | 36 | 60 | USDA |
| *S. cereale cereale* | PI314964 | 7 | AW | Brazil | Cultivar | -22 | -49 | USDA |
| *S. cereale cereale* | PI323450 | 5 | Cfb | Poland | Cultivar | 51 | 19 | USDA |
| *S. cereale cereale* | PI330407 | 6 | BWk | South Africa | Cultivar | -25 | 30 | USDA |
| *S. cereale cereale* | PI330526 | 13 | Cfb | UK | Cultivar | 53 | -2 | USDA |
| *S. cereale cereale* | PI344970 | 12 | Dfb | Bosnia | Landrace | 43 | 19 | USDA |
| *S. cereale cereale* | PI344975 | 17 | Cfb | Montenegro | Landrace | 43 | 19 | USDA |
| *S. cereale cereale* | PI344990 | 11 | Cfb | Serbia | Landrace | 43 | 21 | USDA |
| *S. cereale cereale* | PI345739 | 8 | Cfb | Australia | Cultivar | -35 | 149 | USDA |
| *S. cereale cereale* | PI405812 | 5 | Cfb | Macedonia | Landrace | 42 | 22 | USDA |
| *S. cereale cereale* | PI410534 | 12 | Cfa | Pakistan | Landrace | 34 | 73 | USDA |
| *S. cereale cereale* | PI429371 | 6 | BSk | Iran | Landrace | 36 | 53 | USDA |
| *S. cereale cereale* | PI429373 | 9 | BSk | Iran | Landrace | 36 | 48 | USDA |
| *S. cereale cereale* | PI429377 | 9 | Dsa | Iran | Landrace | 35 | 49 | USDA |
| *S. cereale cereale* | PI430003 | 4 | ET | India | Landrace | 33 | 77 | USDA |
| *S. cereale cereale* | PI436190 | 6 | Cfb | Chile | Cultivar | -40 | -73 | USDA |
| *S. cereale cereale* | PI445977 | 11 | BSh | Israel | Cultivar | 32 | 35 | USDA |
| *S. cereale cereale* | PI445996 | 9 | Cfb | Sweden | Cultivar | 56 | 14 | USDA |
| *S. cereale cereale* | PI446023 | 9 | Cwb | Mexico | Cultivar | 19 | -99 | USDA |
| *S. cereale cereale* | PI446027 | 13 | Cfb | New Zealand | Cultivar | -42 | 174 | USDA |
| *S. cereale cereale* | PI447337 | 4 | BSk | China | Cultivar | 44 | 87 | USDA |
| *S. cereale cereale* | PI535147 | 6 | Am | Yugoslavia | Landrace | 43 | 20 | USDA |
| *S. cereale cereale* | PI543398 | 13 | Aw | Argentina | Cultivar | -35 | -64 | USDA |
| *S. cereale cereale* | PI561793 | 11 | Csa | Turkey | Landrace | 39 | 28 | USDA |
| *S. cereale cereale* | PI561796 | 5 | Cfa | Turkey | Landrace | 41 | 31 | USDA |
| *S. cereale cereale* | PI561797 | 4 | Cfb | Turkey | Landrace | 41 | 32 | USDA |
| *S. cereale cereale* | PI561798 | 9 | Cfb | Turkey | Landrace | 42 | 35 | USDA |
| *S. cereale cereale* | PI561799 | 8 | Cfb | Turkey | Landrace | 42 | 35 | USDA |
| *S. cereale cereale* | PI561802 | 5 | Dsb | Turkey | Landrace | 39 | 42 | USDA |
| *S. cereale cereale* | PI561804 | 6 | Dsb | Turkey | Landrace | 38 | 43 | USDA |
| *S. cereale cereale* | PI561806 | 10 | Dsb | Turkey | Landrace | 40 | 41 | USDA |
| *S. cereale cereale* | PI561809 | 5 | BWk | Pakistan | Landrace | 36 | 74 | USDA |
| *S. cereale cereale* | PI568118 | 1 | Csb | Turkey | Landrace | 40 | 30 | USDA |
| *S. cereale cereale* | PI584782 | 11 | Dfc | Georgia | Landrace | 42 | 46 | USDA |
| *S. cereale cereale* | R1092 | 1 | Cfb | Austria | Cultivar | 48 | 13 | IPK |
| *S. cereale cereale* | R1133 | 1 | Csa | Portugal | Cultivar | 40 | -8 | IPK |
| *S. cereale cereale* | R1148 | 1 | Csa | Turkey | Landrace | 41 | 36 | IPK |
| *S. cereale cereale* | R1151 | 1 | Dfc | Switzerland | Cultivar | 47 | 8 | IPK |
| *S. cereale cereale* | R1480 | 1 | Cfb | Germany | Cultivar | 52 | 9 | IPK |
| *S. cereale cereale* | R1489 | 1 | Cfb | Germany | Cultivar | 51 | 9 | IPK |
| *S. cereale cereale* | R1490 | 1 | Cfb | Germany | Cultivar | 51 | 10 | IPK |
| *S. cereale cereale* | R1653 | 1 | Cfb | Germany | Cultivar | 52 | 11 | IPK |
| *S. cereale cereale* | R1658 | 1 | Dfa | USA | Cultivar | 40 | -99 | IPK |
| *S. cereale cereale* | R1756 | 1 | Dfc | Austria | Cultivar | 47 | 13 | IPK |
| *S. cereale cereale* | R191 | 1 | Cfb | Germany | Cultivar | 50 | 10 | IPK |
| *S. cereale cereale* | R2022 | 1 | Dfc | Finland | Cultivar | 64 | 26 | IPK |
| *S. cereale cereale* | R2199 | 1 | Cfb | Italy | Cultivar | 43 | 13 | IPK |
| *S. cereale cereale* | TR1 | 12 | Csa | Turkey | Landrace | 37 | 29 | Collected |
| *S. cereale cereale* | TR2 | 10 | Dfb | Turkey | Landrace | 41 | 43 | Collected |
| *S. strictum anatolicum* | PI445973 | 12 | Cfa | United States | Wild | 38 | -98 | USDA |
| *S. strictum anatolicum* | R1055 | 1 | Dfa | Armenia | Wild | 41 | 46 | IPK |
| *S. strictum irmanuso* | R858 | 1 | Cfb | Italy | Wild | 44 | 12 | IPK |
| *S. strictum kuprijanovii* | R1053 | 1 | Cfb | Slovenia | Wild | 46 | 15 | IPK |
| *S. strictum kuprijanovii* | R1056 | 1 | Cfa | Azerbaijan | Wild | 41 | 48 | IPK |
| *S. strictum kuprijanovii* | R1151 | 1 | BSk | Kazakhistan | Wild | 48 | 68 | IPK |
| *S. strictum kuprijanovii* | R1154 | 1 | Dfc | Russian Federation | Wild | 57 | 101 | IPK |
| *S. strictum kuprijanovii* | R579 | 1 | Cfa | Azerbaijan | Wild | 41 | 48 | IPK |
| *S. strictum kuprijanovii* | R590 | 1 | Dfc | Russian Federation | Wild | 64 | 104 | IPK |
| *S. strictum strictum* | PI205222 | 10 | Csb | Turkey | Wild | 40 | 31 | USDA |
| *S. strictum strictum* | PI253956 | 2 | Csa | Iraq | Wild | 37 | 43 | USDA |
| *S. strictum strictum* | PI283981 | 2 | Cfb | Hungary | Wild | 47 | 20 | USDA |
| *S. strictum strictum* | PI383757 | 6 | Dsb | Turkey | Wild | 40 | 41 | USDA |
| *S. strictum strictum* | PI401401 | 2 | Csa | Iraq | Wild | 37 | 46 | USDA |
| *S. strictum strictum* | PI401402 | 12 | Csa | Iran | Wild | 33 | 49 | USDA |
| *S. strictum strictum* | PI401404 | 7 | BSk | Iran | Wild | 38 | 46 | USDA |
| *S. strictum strictum* | PI531829 | 10 | Dfb | Armenia | Wild | 41 | 45 | USDA |
| *S. strictum strictum* | PI568257 | 13 | Dfb | Russian Federation | Wild | 60 | 47 | USDA |
| *S. strictum strictum* | R1000 | 1 | Cfb | Italy | Wild | 43 | 12 | IPK |
| *S. strictum strictum* | R1047 | 1 | Dfb | Armenia | Wild | 40 | 45 | IPK |
| *S. strictum strictum* | R914 | 1 | Cfa | Italy | Wild | 42 | 15 | IPK |
| *S. strictum strictum* | R920 | 1 | Csa | Italy | Wild | 42 | 14 | IPK |
| *S. strictum strictum* | R939 | 1 | Csa | Italy | Wild | 42 | 13 | IPK |
| *S. sylvestre* | R1045 | 1 | Cfb | Hungary | Wild | 47 | 19 | IPK |
| *S. sylvestre* | R1046 | 1 | Cfb | Romania | Wild | 46 | 28 | IPK |
| *S. vavilovii* | PI253957 | 13 | Dsb | Afghanistan | Wild | 34 | 68 | USDA |
| *S. vavilovii* | PI284842 | 11 | Cfb | Hungary | Wild | 47 | 20 | USDA |
| *S. vavilovii* | PI573648 | 9 | Dfa | Russian Federation | Wild | 43 | 44 | USDA |
| *S. vavilovii* | PI573649 | 8 | Dsb | Afghanistan | Wild | 35 | 66 | USDA |
| *S. vavilovii* | R1027 | 1 | Cfb | Italy | Wild | 44 | 13 | IPK |
| *S. vavilovii* | R1063 | 1 | Cfb | Poland | Wild | 52 | 21 | IPK |
| *S. vavilovii* | R1125 | 1 | Cfb | Turkey | Wild | 41 | 35 | IPK |
| *S. vavilovii* | R1126 | 1 | Cfb | Turkey | Wild | 41 | 34 | IPK |
| *S. vavilovii* | R2433 | 1 | Unknown | Unknown | Wild |  |  | IPK |
| *S. vavilovii x cereale* | R1064 | 1 | Cfb | Poland | Hybrid | 52 | 20 | IPK |
| *S. vavilovii x cereale* | R1127 | 1 | Csa | Turkey | Hybrid | 38 | 36 | IPK |
| *S. vavilovii x cereale* | R1156 | 1 | Dfc | Russian Federation | Hybrid | 56 | 100 | IPK |
| *S. vavilovii x cereale* | R227 | 1 | Unknown | Unknown | Hybrid |  |  | IPK |
| *S. vavilovii x cereale* | R2432 | 1 | BSk | Afghanistan | Hybrid | 37 | 65 | IPK |
