# Supplementary material for: Phylogeny and genetic structure in the genus Secale
Source: PLoS One. 2018 Jul 19;13(7):e0200825. doi: 10.1371/journal.pone.0200825 (PMC6053196; doi:10.1371/journal.pone.0200825)
Supplement: S2 Table — Chr, Chromosome number; Temp, Annealing Temperature (°C); ref., Reference. (DOCX) [file pone.0200825.s002.docx]

**S2 Table. Molecular markers used in the study.** Chr, Chromosome number; Temp, Annealing Temperature (ºC); ref., Reference.

| **Name** | **Type** | **Length** | **Chr.** | **Temp.** | **Ref.** |
| --- | --- | --- | --- | --- | --- |
| REMS1303 | SSR | 309 | 1R | 55 | Khlestkina et al., 2004 |
| REMS1238 | SSR | 286 | 2R | 60 | Khlestkina et al., 2004 |
| REMS1254 | SSR | 311 | 3R | 65 | Khlestkina et al., 2004 |
| REMS1323 | SSR | 292 | 3R | 65 | Khlestkina et al., 2004 |
| REMS1160 | SSR | 228 | 4R | 60 | Khlestkina et al., 2004 |
| REMS1205 | SSR | 281 | 5R | 62 | Khlestkina et al., 2004 |
| REMS1264 | SSR | 282 | 5R | 56 | Khlestkina et al., 2004 |
| REMS1259 | SSR | 271 | 6R | 52 | Khlestkina et al., 2004 |
| SCM180 | SSR | 158 | 6RL | 65 | Saal and Wricke, 1999 |
| REMS1187 | SSR | 215 | 7R | 60 | Khlestkina et al., 2004 |
| GBS0186 | SNP | 485 | 3R | 63 | Varshney et al., 2005 |
| GBS0456 | SNP | 410 | 4R | 60 | Varshney et al., 2005 |
| GBS0526 | SNP | 674 | 6R | 62 | Varshney et al., 2005 |
| GBS0551 | SNP | 762 | 4R | 63 | Varshney et al., 2005 |
