# Supplementary material for: Phylogeny and genetic structure in the genus Secale
Source: PLoS One. 2018 Jul 19;13(7):e0200825. doi: 10.1371/journal.pone.0200825 (PMC6053196; doi:10.1371/journal.pone.0200825)
Supplement: S3 Table — S. c. afg., S. cereale subsp. afghanicum; S. c. anc., S. cereale subsp. ancestrale; S. c. cereale, S. cereale subsp. cereale; S. c. dighor., S. cereale subsp. dighoricum; S. c. segetale, S. cereale subsp. segetale; S. s. anatol, S. strictum subsp. anatolicum; S. s. irm. S. strictum subsp. irmanuso; S. s. kupr., S. strictum subsp. kuprijanovii; S. s. strictum, S. strictum subsp. strictum; S. sylv., S. sylvestre; S. vav., S. vavilovii. Significant P values are indicated in bold. (DOCX) [file pone.0200825.s003.docx]

**S3 Table. Comparison of pairwise F_ST_ genetic distances between subspecies**. S. c. afg., *S. cereale* subsp. *afghanicum*; S. c. anc., *S. cereale* subsp. ancestrale; S. c. cereale, *S. cereale* subsp. *cereale*; S. c. dighor., *S. cereale* subsp. *dighoricum*; S. c. segetale, *S. cereale* subsp. *segetale;* S. s. anatol, *S. strictum* subsp. *anatolicum*; S. s. irm. *S. strictum* subsp. *irmanuso*; S. s. kupr., *S. strictum* subsp. *kuprijanovii*; S. s. strictum, *S. strictum* subsp. *strictum*; S. sylv., *S. sylvestre*; S. vav., *S. vavilovii.* Significant *P* values are indicated in bold.

| ***S. c. afg.*** | ***S. c*. *anc.*** | ***S. c*. *cereale*** | ***S. c*. *dighor.*** | ***S. c*. *segetale*** | ***S. s*. *anatol.*** | ***S. s*. *irm.*** | ***S. s*. *kupr.*** | ***S. s*. *strictum*** | ***S. sylv.*** | ***S. vav.*** |  |
| --- | --- | --- | --- | --- | --- | --- | --- | --- | --- | --- | --- |
| 0 |  |  |  |  |  |  |  |  |  |  | ***S. c. afg.*** |
| **0.08** | 0 |  |  |  |  |  |  |  |  |  | ***S. c*. *anc.*** |
| **0.06** | 0.03 | 0 |  |  |  |  |  |  |  |  | ***S. c*. *cereale*** |
| **0.39** | **0.37** | **0.33** | 0 |  |  |  |  |  |  |  | ***S. c*. *dighor.*** |
| **0.06** | 0.04 | 0.01 | **0.33** | 0 |  |  |  |  |  |  | ***S. c*. *segetale*** |
| **0.08** | **0.06** | 0.02 | **0.35** | 0.03 | 0 |  |  |  |  |  | ***S. s*. *anatol.*** |
| **0.36** | **0.31** | **0.3** | **0.55** | **0.31** | **0.3** | 0 |  |  |  |  | ***S. s*. *irm.*** |
| **0.09** | **0.05** | 0.02 | **0.36** | 0.04 | 0.04 | **0.32** | 0 |  |  |  | ***S. s*. *kupr.*** |
| **0.06** | 0.04 | 0.02 | **0.35** | 0.01 | 0.03 | **0.29** | 0.03 | 0 |  |  | ***S. s*. *strictum*** |
| **0.18** | **0.12** | **0.1** | **0.45** | **0.11** | **0.09** | **0.32** | **0.1** | **0.09** | 0 |  | ***S. sylv.*** |
| **0.07** | 0.04 | 0.01 | **0.33** | 0.01 | 0.02 | **0.28** | 0.03 | 0.02 | **0.1** | 0 | ***S. vav.*** |
