# Supplementary material for: Phylogeny and genetic structure in the genus Secale
Source: PLoS One. 2018 Jul 19;13(7):e0200825. doi: 10.1371/journal.pone.0200825 (PMC6053196; doi:10.1371/journal.pone.0200825)
Supplement: S4 Table — Am, Tropical monsoon climate; Aw, Savanna; BSh, Hot semi-arid; BSk, Cold semi-arid; BWh, Hot desert; BWk, Cold desert; Cfa, Humid subtropical climate; Cfb,Temperate oceanic climate; Csa, Hot-summer Mediterranean climate; Csb, Warm-summer Mediterranean climate; Cwb, Subtropical highland climate; Dfa, Hot-summer humid continental climate; Dfb, Warm-summer humid continental climate; Dfc, Subarctic climate; Dsa Hot, dry-summer continental climate; Dsb, Warm, dry-summer continental climate; Dwa, Monsoon-influenced hot-summer humid continental climate; ET, Mild tundra climate. Significant P values are indicated in bold. (DOCX) [file pone.0200825.s004.docx]

**S4 Table. Comparison of pairwise F_ST_ genetic distances between climate zone groups.** Am, Tropical monsoon climate; Aw, Savanna; BSh , Hot semi-arid; BSk, Cold semi-arid; BWh, Hot desert; BWk, Cold desert; Cfa, Humid subtropical climate; Cfb,Temperate oceanic climate; Csa, Hot-summer Mediterranean climate; Csb, Warm-summer Mediterranean climate; Cwb, Subtropical highland climate; Dfa, Hot-summer humid continental climate; Dfb, Warm-summer humid continental climate; Dfc, Subarctic climate; Dsa Hot, dry-summer continental climate; Dsb, Warm, dry-summer continental climate; Dwa, Monsoon-influenced hot-summer humid continental climate; ET, Mild tundra climate. Significant *P* values are indicated in bold.

| **Am** | **Aw** | **BSh** | **BSk** | **BWh** | **BWk** | **Cfa** | **Cfb** | **Csa** | **Csb** | **Cwb** | **Dfa** | **Dfb** | **Dfc** | **Dsa** | **Dsb** | **Dwa** | **ET** |  |
| --- | --- | --- | --- | --- | --- | --- | --- | --- | --- | --- | --- | --- | --- | --- | --- | --- | --- | --- |
| 0 |  |  |  |  |  |  |  |  |  |  |  |  |  |  |  |  |  | **Am** |
| **0.13** | 0 |  |  |  |  |  |  |  |  |  |  |  |  |  |  |  |  | **Aw** |
| **0.16** | **0.05** | 0 |  |  |  |  |  |  |  |  |  |  |  |  |  |  |  | **BSh** |
| **0.14** | 0.03 | 0.03 | 0 |  |  |  |  |  |  |  |  |  |  |  |  |  |  | **BSk** |
| **0.17** | **0.08** | **0.05** | 0.04 | 0 |  |  |  |  |  |  |  |  |  |  |  |  |  | **BWh** |
| **0.15** | 0.04 | 0.04 | 0.01 | **0.05** | 0 |  |  |  |  |  |  |  |  |  |  |  |  | **BWk** |
| **0.12** | 0.03 | 0.03 | 0.01 | 0.03 | 0.02 | 0 |  |  |  |  |  |  |  |  |  |  |  | **Cfa** |
| **0.13** | 0.03 | 0.03 | 0.01 | 0.04 | 0.01 | 0.01 | 0 |  |  |  |  |  |  |  |  |  |  | **Cfb** |
| **0.12** | 0.03 | 0.03 | 0.01 | 0.03 | 0.02 | 0.00 | 0.01 | 0 |  |  |  |  |  |  |  |  |  | **Csa** |
| **0.14** | 0.04 | 0.03 | 0.01 | 0.04 | 0.02 | 0.01 | 0.01 | 0.01 | 0 |  |  |  |  |  |  |  |  | **Csb** |
| **0.14** | **0.08** | **0.07** | **0.06** | **0.08** | **0.08** | 0.03 | **0.05** | 0.03 | **0.05** | 0 |  |  |  |  |  |  |  | **Cwb** |
| **0.13** | 0.04 | 0.04 | 0.02 | 0.04 | 0.03 | 0.01 | 0.02 | 0.01 | 0.02 | **0.05** | 0 |  |  |  |  |  |  | **Dfa** |
| **0.13** | 0.03 | 0.03 | 0.01 | 0.03 | 0.03 | 0.01 | 0.01 | 0.01 | 0.01 | 0.04 | 0.01 | 0 |  |  |  |  |  | **Dfb** |
| **0.12** | 0.03 | 0.04 | 0.01 | 0.04 | 0.02 | 0.01 | 0.01 | 0.01 | 0.02 | **0.05** | 0.02 | 0.02 | 0 |  |  |  |  | **Dfc** |
| **0.17** | **0.07** | **0.05** | 0.02 | 0.03 | 0.03 | 0.03 | 0.03 | 0.03 | 0.03 | **0.09** | 0.04 | 0.04 | 0.04 | 0 |  |  |  | **Dsa** |
| **0.14** | 0.04 | 0.04 | 0.01 | 0.04 | 0.02 | 0.01 | 0.01 | 0.01 | 0.01 | 0.04 | 0.02 | 0.01 | 0.02 | 0.03 | 0 |  |  | **Dsb** |
| **0.13** | **0.05** | 0.04 | 0.03 | **0.05** | 0.04 | 0.02 | 0.02 | 0.02 | 0.03 | 0.04 | 0.03 | 0.02 | 0.03 | 0.05 | 0.02 | 0 |  | **Dwa** |
| **0.19** | **0.08** | **0.08** | 0.04 | **0.07** | 0.04 | 0.05 | **0.05** | **0.06** | **0.05** | **0.11** | **0.06** | **0.06** | **0.06** | 0.04 | 0.04 | **0.06** | 0 | **ET** |
