# Supplementary material for: Phylogeny and genetic structure in the genus Secale
Source: PLoS One. 2018 Jul 19;13(7):e0200825. doi: 10.1371/journal.pone.0200825 (PMC6053196; doi:10.1371/journal.pone.0200825)
Supplement: S5 Table — Significant P values are indicated in bold. (DOCX) [file pone.0200825.s005.docx]

**S5 Table. The pairwise F_ST_ values of cultivated rye each geographical region calculated using seven microsatellite markers**. Significant *P* values are indicated in bold.

| **Africa** | **Australia** | **Balkans** | **Caucasus** | **East Asia** | **Europe** | **M. East** | **N. America** | **S. America** | **S. C. Asia** |  |
| --- | --- | --- | --- | --- | --- | --- | --- | --- | --- | --- |
| 0 |  |  |  |  |  |  |  |  |  | **Africa** |
| **0.08** | 0.00 |  |  |  |  |  |  |  |  | **Australia** |
| **0.08** | 0.02 | 0.00 |  |  |  |  |  |  |  | **Balkans** |
| **0.09** | 0.04 | 0.02 | 0.00 |  |  |  |  |  |  | **Caucasus** |
| **0.06** | 0.03 | 0.03 | 0.05 | 0.00 |  |  |  |  |  | **East Asia** |
| **0.09** | 0.02 | 0.01 | 0.02 | 0.04 | 0.00 |  |  |  |  | **Europe** |
| **0.08** | 0.02 | 0.01 | 0.02 | 0.03 | 0.01 | 0.00 |  |  |  | **M. East** |
| **0.07** | 0.02 | 0.02 | 0.03 | 0.02 | 0.03 | 0.02 | 0.00 |  |  | **N. America** |
| **0.06** | 0.04 | 0.02 | 0.03 | 0.03 | 0.03 | 0.03 | 0.02 | 0.00 |  | **S. America** |
| **0.10** | 0.02 | 0.02 | 0.02 | 0.03 | 0.01 | 0.01 | 0.02 | 0.04 | 0.00 | **S. C. Asia** |
